# Advances in Chitin and Chitosan Science

**DOI:** 10.3390/molecules26061805

**Published:** 2021-03-23

**Authors:** Massimiliano Fenice, Susanna Gorrasi

**Affiliations:** Dipartimento di Ecologia e Biologia, University of Tuscia, 01100 Viterbo, Italy; gorrasi@unitus.it

Chitin is among the most abundant natural polysaccharides. This linear homopolymer of *N*-Acetyl-d-glucosamine and its deacetylated form (chitosan) have various biological and physiochemical properties: biodegradability, biocompatibility, and bioactivity. Chitin, chitosan, and their derivative are subjects of many studies leading to broad range applications in agriculture, biotechnology, and medicine: their use span from antimicrobial compounds to substrates for bio-energy production [1,2]. In addition, the various enzymes involved in their degradation and/or modification have received large attention, having important ecological roles and wide range of uses (from the production of specific derivatives to chitin/chitosan rich materials degradation and biological pest control) [3,4]. Among these enzymes, those produced by microorganisms are most interesting for their wide diffusion and easy production for possible applications. Bacteria and fungi from the different environments (from Antarctica soil to various marine environments) are considered the best sources of these enzymes [4,5,6,7].

Thus, the investigation of these polysaccharides and the related enzymes continue involving numerous scientists gathering in international societies and actively participating in international conferences. Many basic and application works have been done in the last decades to depict structure and potentiality of these biopolymers. Nevertheless, the number of new studies devoted to these topics is still high, indicating a constant vivid interest regarding chitin and chitosan science, which is also demonstrated by specific books and various recent special issues in different Journals.

The Special Issue “Advances in Chitin and Chitosan Science” was aimed to give room to a broad platform for the diffusion of the most recent studies regarding chitin, chitosan, and related enzymes. Major topics were: chitin/chitosan chemistry and biochemistry; production and applications of chitin/chitosan and derivatives; bio-synthesis and bio-degradation of chitin/chitosan; and chitinolytic organisms and their applications. Thirteen high-quality papers, two review papers and one short report, describing new aspects of chitin and chitosan science, were published. The Editorial Committee accepted only particularly innovative papers, covering the various aspects of the issue, as reported by the following abstracts: 

-*Obtaining and Characterization of the PLA/Chitosan Foams with Antimicrobial Properties Achieved by the Emulsification Combined with the Dissolution of Chitosan by CO2 Saturation*: A new method of obtaining functional foam material was proposed. The materials were created by mixing a poly-lactic acid (PLA) chloroform solution, chitosan (CS) dissolved in CO2-saturated water and polyethylene glycol (PEG). Freeze-drying was used for solvent removal. The composite foams were characterized for their structural, thermal (DSC), functional, and biological properties. Chitosan in the composites was a component for obtaining their foamed form with 7.4–22.7 times lower density compared to the neat PLA and high porosity also confirmed by SEM. Foams had a hardness in the range of 70–440 kPa. The FT-IR analysis confirmed no new chemical bonds between the sponge ingredients. Other results showed low sorption capacity (2.5–7.2 g/g) and solubility of materials (<0.2%). The foams had the lower T_g_ and improved crystallization ability compared to neat PLA. The addition of chitosan provides bacteriostatic/bactericidal properties against Escherichia coli and Staphylococcus aureus. Biocompatibility studies showed that the materials were not cytotoxic to the L929 cell line [8].

-*Dual Insecticidal Effects of Adenanthera pavonina Kunitz-Type Inhibitor on Plodia interpunctella is Mediated by Digestive Enzymes Inhibition and Chitin-Binding Properties*. The Indianmeal moth, P. interpunctella, is among most damaging pests of stored products. We investigated the insecticidal properties of ApKTI, a Kunitz trypsin inhibitor from A. pavonina seeds, against P. interpunctella larvae through bioassays with artificial diet. ApKTI-fed larvae showed reduction of up to 88% on weight and 75% in survival. Trypsin enzymes extracted from P. interpunctella larvae were inhibited by ApKTI, which also demonstrated chitin-binding capacity. Kinetic studies revealed a non-competitive inhibition mechanism for trypsin, which were further corroborated by molecular docking studies. Furthermore, ApKTI exhibits a hydrophobic pocket near the reactive site loop, probably involved in chitin interactions. Results suggested that ApKTI insecticidal activity for P. interpunctella larvae involves a dual and promiscuous biding mechanisms to two different targets. Both processes might impair the larval digestive process, leading to death before the pupal stage. Further studies are encouraged using ApKTI as a biotechnological tool to control insect pests in field conditions [9].

-*Development-Disrupting Chitin Synthesis Inhibitor, Novaluron, Reprogramming the Chitin Degradation Mechanism of Red Palm Weevils*. Disruption in chitin regulation by using chitin synthesis inhibitor (novaluron) was investigated to understand the biological activity of chitinase in red palm weevils (RPW), a date palm invasive pest in the Middle East. Novaluron impact against ninth-instar RPW larvae was examined by dose-mortality response bioassays, nutritional indices, and expression patterns of chitinase genes. Bioassays revealed dose-dependent mortality response of RPW larvae with LD_50_ of 14.77 ppm of novaluron. Dietary growth analysis performed using different doses of novaluron exhibited very high reduction in their indexes such as Efficacy of Conversion of Digested Food (82.38%) and Efficacy of Conversion of Ingested Food (74.27%), compared with control treatment. Transcriptomic analysis of RPW larvae characterized numerous genes involved in chitin degradation. However, quantitative expression patterns of these genes in response to novaluron-fed larvae revealed tissue-specific time-dependent expression patterns. We recorded overexpression of all genes from mid-gut tissues. Growth retarding, chitin remodeling and larvicidal potential suggest novaluron as a promising alternate for Rhynchophorus ferrugineus management [10].

*-Efficient Removal of Copper Ion from Wastewater Using a Stable Chitosan Gel Material.* Gel adsorption is an efficient method to remove metal ion. In this study, a functional chitosan gel material (FCG) was synthesized, and its structure was detected by different physicochemical techniques. The as-prepared FCG was stable in acid and alkaline media and showed excellent adsorption properties for Cu^2+^ capture from aqueous solution. The FCG maximum adsorption capacity was 76.4 mg/g for Cu^2+^. The kinetic adsorption data fits the Langmuir isotherm, and experimental isotherm data follows the pseudo-second-order kinetic model well, suggesting that it is a monolayer and the rate-limiting step is the physical adsorption. The separation factor (*R*_L_) for Langmuir and the 1/*n* value for Freundlich isotherm show that the Cu^2+^ is favourably adsorbed by FCG. The negative values of enthalpy and Gibbs free energy indicate that the adsorption process is exothermic and spontaneous in nature. Fourier transform infrared spectroscopy and X-ray photoelectron spectroscopy analysis of FCG, before and after adsorption, further revealed the mechanism of Cu^2+^ adsorption. Further desorption and reuse experiments show that FCG still retains 96% of the original adsorption following the fifth adsorption–desorption cycle. Results indicate that FCG is a promising recyclable adsorbent for the removal of Cu^2+^ from aqueous solution [11].

*-Spider Chitin: An Ultrafast Microwave-Assisted Method for Chitin Isolation from Caribena versicolor Spider Molt Cuticle*. Chitin, a fundamental polysaccharide in invertebrate skeletons, continues to be actively investigated, especially with respect to new sources and the development of effective extraction methods. Recent attention was focused on crustaceans and sponges; however, the potential of spiders as an alternative source of tubular chitin was overlooked. This work focused on chitin from up to 12 cm-large Theraphosidae spiders, popularly known as tarantulas or bird-eating spiders, which “lose” large quantities of cuticles during molt. Here, we present a highly effective method for chitin isolation from *Caribena versicolor* spider molt cuticle, its identification and characterization using modern analytical methods. We suggest that the tube-like molt cuticle of this spider can serve as a naturally prefabricated and renewable source of tubular chitin, with high potential in technology and biomedicine applications [12].

*-The Importance of Reaction Conditions on the Chemical Structure of N,O-Acylated Chitosan Derivatives*. The structure of acylated chitosan derivatives strongly determines the properties of obtained products, influencing their hydrodynamic and their solubility or self-assembly susceptibility. In this work, the significance of slight changes in acylation conditions on the structure and properties of the products is discussed. Various chitosan-acylated derivatives was synthesized by varying reaction conditions in a two-step process. As reaction media, two diluted acid solutions (acetic acid and hydrochloric acid) and two coupling systems (1-ethyl-3-(3-dimethyl-aminopropyl)-1-carbodiimide hydrochloride (EDC) and *N*–hydroxysulfosuccinimide (EDC/NHS)) were used. The derivative chemical structure was studied by two spectroscopic methods (infrared and nuclear magnetic resonance spectroscopy) to analyze the system preference towards *N*- or *O*-acylation reactions, depending on the synthesis conditions used. The results obtained from advanced ^1^H-^13^C HMQC spectra emphasized the challenge of achieving a selective acylation reaction path. The study of derivative molecular weight and solution behavior revealed that even slight changes in their chemical structure have an important influence on their final properties. Therefore, an exact knowledge of the obtained structure of derivatives is essential to achieve reaction reproducibility and to target the application [13].

*-Chitosan-Based Bioactive Hemostatic Agents with Antibacterial Properties—Synthesis and Characterization*. Massive blood loss is responsible for numerous deaths. Haemorrhage may occur on the battlefield, at home or during surgery. Commercial biomaterials may be insufficient to deal with excessive bleeding. Therefore, novel, highly efficient haemostatic agents must be developed. Aim of this research was to obtain a new type of biocompatible chitosan-based haemostatic agents with increased haemostatic properties. The biomaterials were quickly and efficiently obtained under microwave radiation using l-aspartic and l-glutamic acid as crosslinking agents with no use of acetic acid. Products chemical structure were investigated by FT-IR, confirming a crosslinking process through the formation of amide bonds. Their high porosity (>90%) and low density (<0.08 g/cm^3^) were confirmed. The aerogels were also studied over their water vapor permeability and antioxidant activity. Biomaterials were biodegradable by human lysozyme. All samples had excellent hemostatic properties in contact with human blood due to the platelet activation, confirmed by blood clotting tests. SEM microphotographs showed the blood cells adherence to the biomaterials’ surface. Moreover, they were biocompatible with human dermal fibroblasts and had superior antibacterial properties against both *S. aureus* and *E. coli*. The results showed that proposed chitosan-based hemostatic agents have great potential as a hemostatic product and may be applied under sterile and contaminated conditions, by both medicals and individuals [14].

*-Treatment of Contaminated Groundwater* via *Arsenate Removal Using Chitosan-Coated Bentonite.* In this research, treatment of contaminated groundwater via adsorption of As(V) with an initial concentration of 50.99 µg/L using chitosan-coated bentonite (CCB) was investigated. The effect of adsorbent mass, temperature, and contact time on the removal efficiency was examined. Adsorption data was evaluated using various isotherm models. Isotherm study showed that the Langmuir model best correlates (*R*^2^ > 0.9899; *χ*^2^ ≤ 0.91; *RMSE* ≤ 4.87) with the experimental data. Kinetics studies revealed that pseudo-second order equation adequately describes the experimental data (*R*^2^ ≥ 0.9951; *χ*^2^ ≤ 8.33; *RMSE* ≤ 4.31) where equilibrium was attained after 60 min. Thermodynamics study shows that the As(V) adsorption is non-spontaneous (Δ*G*^0^ ≥ 0) and endothermic (Δ*H*^0^ = 8.31 J/mol) that would result in an increase in randomness (Δ*S*^0^ = 29.10 kJ/mol·K) within the CCB-solution interface. FT-IR analysis reveals that hydroxyl and amino groups are involved in the adsorption of As(V) from groundwater. Our results serve as a tool to determine whether CCB is an environmentally safe and cost effective material that could be utilized in a permeable reactive barrier system for the remediation of As(V) from contaminated groundwater [15].

*-Gallic Acid-Chitosan Conjugate Inhibits the Formation of Calcium Oxalate Crystals.* It has recently been shown that chitosan (Chit) induces the formation of calcium oxalate (CaOx) crystals, which are mainly responsible for the appearance of kidney stones, and this might limit the use of Chit in vivo. Here, Chit was conjugated with gallic acid (Chit-Gal) to decrease the formation of CaOx crystal. Conjugation was confirmed by FTIR and NMR analyses. Chit-Gal contains 10.2 ± 1.5 mg GA per g of Chit. Compared to the control, Chit increased the number of crystals by six-fold, mainly in the number of monohydrated CaOx crystals, the most harmful crystals. In addition, Chit increased the zeta potential (ζ) of crystals by three-fold, indicating that Chit was associated with the crystals. These alterations were abolished when Chit-gal was used in these tests. As oxidative stress is related to renal calculus formation, Chit and Chit-Gal were also evaluated as antioxidants using total antioxidant Capacity (TAC), reducing power, ferrous and copper chelation tests. Chit-gal was more efficient antioxidant agent in TAC (2 times), in ferrous chelation (90 times), and in reducing Power (5 times) than Chit. Overall, Chit-gal has higher antioxidant activity than Chit, does not induce the formation of CaOx crystals. Thus, Chit-Gal has potential to be used as a chit substitute [16].

*-Microbial Corrosion Resistance and Antibacterial Property of Electrodeposited Zn–Ni–Chitosan Coatings.* Microbial corrosion is a universal phenomenon in salt water media such as seawater and wastewater environments. As a kind of efficient protective metal coating for steel, the damage of the Zn–Ni alloy coating accelerates under microbial corrosive conditions. To solve this problem, chitosan, considered a natural product with high antibacterial efficiency, was added to Zn–Ni electrolytes as a functional ingredient of electrodeposited Zn–Ni–chitosan coatings. The addition of chitosan significantly and negatively shifted the electrodeposition potentials and influenced Ni contents, phase composition, and surface morphologies. By exposing the coatings in sulphate-reducing bacteria (SRB) medium, the microbial corrosion resistance was investigated. Compared to the Zn–Ni alloy coating, Zn–Ni–chitosan coatings showed obvious inhibiting effects on SRB and the corrosion rates of these coatings were mitigated to some degree. Further research on the coatings immersed in an *E. coli*-suspended phosphate buffer saline medium showed that the bacteria attachment on the coating surface was effectively reduced, indicating enhanced antibacterial properties. The Zn–Ni–chitosan coatings showed remarkably enhanced anticorrosive and antibacterial properties. [17].

*-High Production of Chitinolytic Activity in Halophilic Conditions by a New Marine Strain of Clonostachys rosea.* Twenty-eight fungal strains were isolated from different natural marine substrates and plate screened for their production of chitinolytic activity. The two best producers, *Trichoderma lixii* IG127 and *Clonostachys rosea* IG119, were screened in shaken cultures in media containing 1% colloidal chitin, 1% yeast nitrogen base (YNB) and 38‰ NaCl, for their ability to produce chitinolytic enzymes under halophilic conditions. Also, they were tested for optimal growth conditions with respect to pH, salinity and temperature. The *Trichoderma* strain was a slight halotolerant fungus, while *C. rosea* was a halophilic marine fungus, being its optimal growth conditions coherent for life in the marine environment (pH 8.0, salinity 38‰). Due to its high and relatively fast activity (258 U/L after 192 h) and halophilic behaviour (growth 0–160‰ salinity), *C. rosea* was selected for further studies. In view of industrial applications, its medium for chitinolytic enzyme production was optimized by Response Surface Methodology using 1% colloidal chitin and different concentrations of corn step liquor (CSL) and YNB (0–0.5%). Maximum activity (394 U/L) and maximum productivity (3.3 U/Lh) under optimized conditions were recorded after 120 h on medium containing 0.47% CSL and 0.37% YNB. This was the first study demonstrating high chitinolytic activity in a marine strain of *C. rosea* [18].

-*Cross-Linking Chitosan into Hydroxypropyl methylcellulose for the Preparation of Neem Oil Coating for Postharvest Storage of Pitaya (Stenocereus pruinosus)*. The market trend for pitaya fruits is increasing, although its post-harvesting preservation is challenging due to microbial decay, dehydration, and oxidation. In this work, the application of antimicrobial chitosan-based coatings achieved successful postharvest preservation of pitaya during storage at 10 ± 2 °C with a relative humidity of 80 ± 5%. The solution of cross-linked chitosan with hydroxypropyl methylcellulose with entrapped Neem oil (16 g·L^−1^) displayed best postharvest fruit characteristics. The reduction of physiological weight loss and fungal contamination, with an increased redness index and release of azadirachtin from the microencapsulated oil, resulted in up to a 15-day shelf life. This postharvest procedure has the potential to increase commercial exploitation of fresh pitaya, owing to its good taste and high content of antioxidants [19].

-*Demethoxycurcumin-Loaded Chitosan Nanoparticle Downregulates DNA Repair Pathway to Improve Cisplatin-Induced Apoptosis in Non-Small Cell Lung Cancer*. Demethoxycurcumin (DMC), through a self-assembled amphiphilic carbomethyl-hexanoyl chitosan (CHC) nanomatrix was successfully developed and used as a therapeutic approach to inhibit cisplatin-induced drug resistance by suppressing excision repair cross-complementary 1 (ERCC1) in non-small cell lung carcinoma cells (NSCLC). Previously, DMC significantly inhibited on-target cisplatin resistance protein, ERCC1, via PI3K-Akt-snail pathways in NSCLC. However, low water solubility and bioavailability of DMC causes systemic elimination and prevents its clinical application. To increase its bioavailability and targeting capacity toward cancer cells, a DMC-polyvinylpyrrolidone core phase was prepared and encapsulated in a CHC shell to form DMC-loaded core-shell hydrogel nanoparticles (DMC-CHC NPs). We aimed understanding whether DMC-CHC NPs efficiently potentiate cisplatin-induced apoptosis through downregulation of ERCC1 in NSCLC. DMC-CHC NPs displayed good cellular uptake efficiency. Dissolved in water, DMC-CHC NPs showed comparable cytotoxic potency with free DMC. A sulforhodamine B (SRB) assay indicated that DMC-CHC NPs significantly increased cisplatin-induced cytotoxicity by highly efficient intracellular delivery of the encapsulated DMC. A combination of DMC-CHC NPs and cisplatin significantly inhibited on-target cisplatin resistance protein, ERCC1, via the PI3K-Akt pathway. Also, this combination treatment markedly increased the post-target cisplatin resistance pathway including Bax, and cytochrome c expressions. Thymidine phosphorylase (TP) was also highly inhibited by the combination treatment. The results suggested that enhancement of the cytotoxicity to cisplatin via administration of DMC-CHC NPs was mediated by down-regulation of the expression of TP, and ERCC1, regulated via the PI3K-Akt pathway [20].

*-Glycol Chitosan: A Water-Soluble Polymer for Cell Imaging and Drug Delivery (review).* Glycol chitosan (GC), a water-soluble chitosan derivative with hydrophilic ethylene glycol branches, has both hydrophobic segments for the encapsulation of various drugs and reactive functional groups for facile chemical modifications. Over the past two decades, various molecules were physically encapsulated within or chemically conjugated with GC and its derivatives to construct a wide range of functional biomaterials. This review summarizes the recent advances of GC-based materials in cell surface labelling, multimodal tumor imaging, and encapsulation/delivery of drugs (including chemotherapeutics, photosensitizers, nucleic acids, and antimicrobial agents) against cancers and microbial infections. Besides, different strategies for GC modifications are highlighted to show how to provide GC and its derivatives with desirable properties in therapy. In addition, the promises and challenges of the GC-derived biomaterials were discussed [21].

*-Chitinases as Food Allergens (review).* Food allergies originate from adverse immune reactions to some food components. Ingestion of food allergens can cause effects of varying severity, from mild itching to severe anaphylaxis reactions. Currently there are neither clues to predict the allergenic potency of a molecule, nor available cures for food allergies. Cutting-edge research on allergens is aimed at increasing information on their diffusion and understanding structure-allergenicity relationships. In this context, purified recombinant allergens are valuable tools for advances in diagnostic and immunotherapeutic fields. Chitinases are a group of allergens often found in plant fruits, but also in edible insects. They are classified into different classes for which structural analyses and identification of epitopes have been only partially carried out. Also, their presence in common allergen databases is not complete. In this review we provide a summary of the identified food allergenic chitinases, their main structural characteristics, and a clear division in different classes [22].

*-Can we make Chitosan by Enzymatic Deacetylation of Chitin? (brief report).* Chitin, an insoluble linear polymer of β-1,4-*N*-acetyl-d-glucosamine (GlcNAc; A), can be converted to chitosan, a soluble heteropolymer of GlcNAc and d-glucosamine (GlcN; D) residues, by partial deacetylation. In nature, deacetylation of chitin is catalyzed by chitin deacetylases (CDA) and it has been proposed that CDAs could be used to produce chitosan. In this work, we show that CDAs can remove up to approximately 10% of *N*-acetyl groups from two different (α and β) chitin nanofibers, but cannot produce chitosan [23].

## Data Availability

not applicable.
